# Should we reframe how we think about physical activity and sedentary behaviour measurement? Validity and reliability reconsidered

**DOI:** 10.1186/s12966-016-0351-4

**Published:** 2016-03-01

**Authors:** Paul Kelly, Claire Fitzsimons, Graham Baker

**Affiliations:** Physical Activity for Health Research Centre, Institute for Sport, Physical Education and Health Sciences, University of Edinburgh, Edinburgh, UK

**Keywords:** Physical activity, Sedentary behaviour, Measurement, Validity, Reliability, Framework

## Abstract

**Background:**

The measurement of physical activity (PA) and sedentary behaviour (SB) is fundamental to health related research, policy, and practice but there are well known challenges to these measurements. Within the academic literature, the terms “validity” and “reliability” are frequently used when discussing PA and SB measurement to reassure the reader that they can trust the evidence.

**Discussion:**

In this paper we argue that a lack of consensus about the best way to define, assess, or utilize the concepts of validity and reliability has led to inconsistencies and confusion within the PA and SB evidence base. Where possible we propose theoretical examples and solutions. Moreover we present an overarching framework (The Edinburgh Framework) which we believe will provide a process or pathway to help researchers and practitioners consider validity and reliability in a standardized way.

**Conclusion:**

Further work is required to identify all necessary and available solutions and generate consensus in our field to develop the Edinburgh Framework into a useful practical resource. We envisage that ultimately the proposed framework will benefit research, practice, policy, and teaching. We welcome critique, rebuttal, comment, and discussion on all ideas presented.

## Background

*This Debate Paper was originally delivered by Paul Kelly as an Invited Early Career Presentation at the ISBNPA Conference in Edinburgh, Scotland on 4th June 2015.*

The measurement of physical activity (PA) and sedentary behaviour (SB) is fundamental to research, policy and practice; whether monitoring population trends, understanding sub-populations and high-risk groups, assessing correlates and determinants, or testing intervention effects. The challenges of measurement are well known, and the limitations of different methods often described. The terms “*validity*” and “*reliability*” (see Table 1 for definitions of key terms) are therefore frequently found in the academic literature. They are widely used in studies examining or using these methods to reassure the reader that they can trust the results. However, in PA and SB measurement there is a lack of consensus about the best way to define, assess and utilize these concepts.

**Table 1:** Description of terms for validity and reliability in PA and SB measurement

*Validity *– the extent to which a measurement is representative of the true scientific value; taking “true” to mean an exact representation of what happened, free from all possible sources of error or bias.	
*Test validity* (*or Construct validity*) – a combined assessment of face, content, and [concurrent, convergent or criterion]validity for your measure within the desired or utilised study population.	
Face validity	The extent to which a measure looks like it will, or appears to, provide the desired information. Assessed by expert consensus and theoretical consideration.
	Likewise for the proposed data processing and generation of outcome variables. Assessed by expert consensus and theoretical consideration.
Content validity	The extent to which a measure covers all aspects of the intended behavioural or physiological domains or dimensions (see Fig. 1). Assessed by examination of domain or dimension of interest.
	Likewise for the proposed data processing and generation of outcome variables.
Convergent validity	The extent of the agreement with another (non-criterion) measure that should assess the same PA or SB parameter based on face and content validity. Assessed quantitatively.
	Useful when the criterion is very resource intensive.
	This approach also allows assessment of whether the measures can be used interchangeably, or the data from the two measures pooled or otherwise compared.
Criterion validity	The extent of the agreement between a measure and another already held as being a criterion or gold standard. Assessed quantitatively. Called absolute validity when compared to measure known to provide perfectly true values.
Concurrent validity	Assessment of convergent or criterion validity when measures taken at same time.
Predictive validity	Assessment of convergent or criterion validity when measures taken at different times.
*Experimental validity *– a combined assessment of internal and external validity to determine whether conclusions drawn from the data are free from bias and generalizable to wider populations.	
Internal validity	The extent to which conclusions drawn from the experimental data are free from confounding issues which cause bias such as reactivity and missing data; similar to methodological quality. Assessed by examination of relevant issues.
External validity	The extent to which conclusions drawn from the data are generalizable to the wider populations. Assessed by examination of age, sex, ethnic origin, socio-economic status, etc., of study sample.
	This could be assessed by a theoretical justification or empirical demonstration such as field testing and small scale “proof of concept” studies. These should assess participant feedback (e.g. satisfaction and burden) as well as data issues (e.g. can meaningful information be produced in reasonable time frames?)
*Reliability* – the extent to which a tool gives measurements that are consistent, stable, and repeatable.	
Test-retest reliability	The extent to which test scores are consistent from one test administration to the next; keeping as many conditions (e.g. researcher, timing, preparation, etc.) as possible unchanged. Assessed quantitatively.
	This estimate incorporates any factors that cannot be controlled e.g. intra-rater reliability, behaviour change, etc.
Inter/intra-rater reliability	The extent to which test scores are consistent when measurements are taken by different people using the same methods (inter-rater) or at different times by the same person (inter-rater). Assessed quantitatively.
Inter/intra-instrument reliability	The extent to which test scores are consistent when measurements of the same thing are taken by different versions of the same instrument (inter-instrument) or repeatedly by the same version of an instrument (intra-instrument). Assessed quantitatively.
Behavioural reliability	The extent to which stability in behaviour has been considered when assessing other aspects of reliability.

Note: We are not attempting to deliberately re-define any term here; if we use one here that you think we have described incorrectly we suggest this is more evidence for non-standard use of terms and further justification for the need of this framework. Multiple sources used

A multitude of approaches have been taken leading to an incorrect perception of the strengths and weaknesses of different methods and a false hierarchy of measures. This limits the extent to which we can compare findings from different validity and reliability studies. It has also led to inappropriate use of measures and misperception in the PA and SB evidence.

In this paper we present the following for debate: first a proposition that PA and SB research needs a comprehensive framework for considering the different aspects of measurement validity and reliability based on a series of inter-related arguments; and second we provide a draft version of such a framework given the authors’ belief that this is indeed warranted. We welcome critique, rebuttal, comment, and discussion on all ideas presented below.

## Discussion

### Part 1: why do we need a measurement framework for Physical Activity and Sedentary Behaviour?

#### Physical Activity and Sedentary Behaviour are not single, unidimensional constructs, but validity and reliability statements rarely acknowledge this

**Fig. 1:**
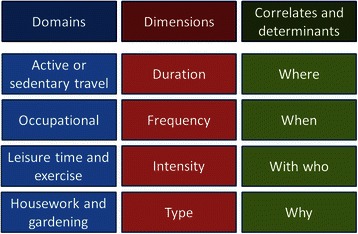
Domains, dimensions, and correlates and determinants of PA and SB. We use this figure to discuss the different ways these behaviours can be described or characterized. It is not meant to be exhaustive, and some may take issue with how we have used ‘determinants’. When considering sedentary behaviour posture may require its own box. Source: PAHRC teaching materials (MSc Physical Activity for Health)

PA and SB are multi-faceted; they can be described by multiple domains, dimensions, and correlates or determinants, with many more sub-groups within each (see Fig. 1). Rowe describes PA as “a somewhat unique construct in kinesiology in that it incorporates behavioural, physiological and biomechanical principles” [1]. Thompson et al., recently argued that “no single metric will reflect an individual’s physical activity adequately because multiple biologically important dimensions are independent and unrelated” [2]. SB is a similarly complex construct, sharing many of the characteristics of PA but also being described by other facets such as posture, status or associated behaviour [3, 4].

However, claims of validity or reliability are often based on a single aspect such as duration, frequency, or intensity; or a composite outcome such as physical activity energy expenditure (PAEE) or MET.hours expended [5]. This means that to simply state a PA or SB measure is valid or reliable misses the crucial information of what aspect it is valid or reliable for (see Box 1).

There has been much work considering the various methodological challenges of measuring PA and SB, and the limitations and advantages have been well reviewed when considering what instrument or measure to select [1, 6–8]. However, many studies discuss validity and reliability without considering the complexity of PA or SB. The work by Rowe et al., which presents an assessment of “accuracy of different instruments for estimating different physical activity dimensions” [1] is an exception rather than the rule.

These considerations of “validity for purpose” would normally be covered by assessment of *face validity* and *content validity* [9]. Unfortunately these assessments are often omitted in favour of *concurrent* or *criterion validity* (see Table 1) [10, 11] despite previous calls to identify measurement purpose as a first step [12]. This means statements of validity and reliability can lack meaning or relevance. Furthermore this leads to claims of criterion validity when the comparator is in no sense a criterion for the domain or dimension of relevance.

We propose that an agreed framework emphasising a focus on the complexity of PA and SB, and specifying what the measure was valid for, would promote better understanding of measurement strengths and weaknesses.

#### Our field has created a situation where doubly labelled water is considered the gold standard for PA measurement

Many studies refer to doubly labelled water (DLW) as the “gold standard” in PA measurement, or that a measure has been “validated” against DLW giving it “validity”. However, if the validity of DLW was assessed against our proposed framework we would quickly conclude that it was a very good measure of total energy expenditure but a poor measure of frequency, type, intensity, or duration. The validity of DLW clearly depends on the purpose of the measurement. The implication of this is that when studies claim “criterion validity” by comparison to DLW, this cannot be the case unless the primary purpose of their measure is also attempting to assess PAEE. The approach of using DLW to validate PA measures, often in nutrition focussed contexts, leads to over-emphasis on energy balance when considering these behaviours despite the varied aspects discussed in point 1.

Depending on the PA or SB aspect of interest there will be different “gold standards”. For behaviours such as walking, sitting, or sports it may be direct researcher observation; for total activity related movement it may be wrist worn accelerometers; for cycling it may be bicycle mounted GPS devices; or for physiological response it may be heart rate monitors. An agreed framework that emphasised face and content validity would allow us to place the correct approaches as the “gold standard” dependent on purpose; this in turn will allow for more appropriate selection of comparators when assessing the validity and reliability of new measures.

The failure to differentiate “gold standard” approaches through appropriate consideration of “validity for purpose” by domain and dimension has led to two apparent and related problems; (1) it continues to drive the quasi-physiological reporting of PA or SB, e.g., studies that say they are assessing behaviour but in reality convert to energy expenditure so they can compare to DLW; and (2) it has contributed to the adoption of a false hierarchy in PA and SB measurement.

We will consider the false hierarchy problem in point 3, below. With respect to quasi-physiological reporting, by prioritising DLW as the gold standard we implicitly place energy expenditure at the top of the “hierarchy of desired outcomes”. While total PAEE is undoubtedly an important metric that reveals useful information, there are clearly many other study designs that are not dependent on this outcome (see Box 2). Thompson et al., warn against trying to reflect an individual’s PA using a single metric [2] and this warning should similarly apply to energy expenditure.

It is not so clear that the field of SB research holds DLW in the same esteem, and studies often refer to direct observation or inclinometers as the gold standard. It is worth noting that currently SB is defined by an energy expenditure threshold (≤1.5 METs while in a siting or reclining posture); that posture is also included is a promising sign that the complexity of the behaviour is considered important [3].

#### The lack of an agreed validity and reliability framework has led to a false measurement hierarchy

This point builds from the previous idea that DLW is held as the gold standard, “most valid”, measure of PA without enough scrutiny. This drives how we think about all other measures; DLW is best, therefore other measures should be judged on their agreement with DLW. Subjective and objective measures are converted into energy based physiological metrics as this is the metric of the assumed gold standard (DLW). Generally, neither shows great statistical agreement [13, 14] but objective measures usually fare better and that leads to the assumption that “objective measurement is good, subjective measurement is bad” (often with the disclaimer that subjective measures are at least cheaper and more scalable and that is why they are used in many cases). We concur with Troiano et al., who report that low correlations between self-report and accelerometer assessed PA are because the methods are “distinct” and “not equivalent” [15].

**Fig. 2:**
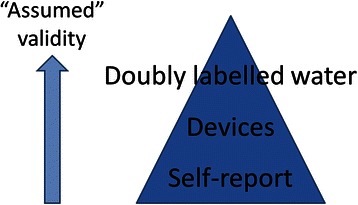
A false hierarchy for PA and SB measurement when considering anything other than PAEE. Source: PAHRC teaching materials (MSc Physical Activity for Health)

We present the “false hierarchy” as our interpretation of prevailing sentiment (see Fig. 2). Perhaps future research should test if our community indeed views DLW and measurement in this way. Improved appraisal of validity and reliability could challenge this hierarchy for outcomes such as cycling frequency in PA, or occupational sitting in SB.

Another problematic assumption with this hierarchy is as follows: accelerometers have been validated against DLW so they are suitable to validate (or more often invalidate) other measures. If the arguments above are accepted, this is clearly a false approach; measures should be validated against the best available measure of that domain or dimension, not always DLW or one that compares well against DLW (see Box 3). This has led to inappropriate claims of *criterion validity*; the criterion has to be justifiably a criterion and our proposed framework would give a structure to the process of this justification. Comparing a self-report measure to an accelerometer may result in *criterion validity* when the purpose is to assess total movement; but it may result in *concurrent validity* when the purpose is to measure bouts of walking especially when domain is important. This will influence how we view validity findings, and in turn how we interpret evidence from studies using these measures. An additional consideration is measures that are multi-dimensional, for example a self-report or device measure that is assessing duration, frequency and location. In this case multiple types of validity evidence will be required, from a variety of comparators or criterions, possibly including direct observation, wearable devices, and existing self-report measures. Our framework would ask the researcher to consider the purpose of the measure and would provide a more nuanced and useful reflection of validity and reliability.

#### Validity and reliability are often reported as single statistics that do not tell us enough

This is perhaps partly because we are tempted towards quantifiable statistics like *r*, *p* and alpha values, over subjective and theoretical assessments of content and face validity. Lacking meaning and context, these statistics however tell us nothing about the extent to which considerations of purpose, domain, and dimension were used to inform the comparison. As described above, a low *r* value could be the expected result of comparing a subjective measure of bouts of walking for leisure to an objective measure of hip movement; it tells us more about the validity of the comparison than about the data from either measure.

There is also an issue about the number and variety of statistical approaches available for comparisons. This is not the place to discuss the merits of different approaches or tests but debates about Bland-Altman tests and regression analysis will be familiar to many [16, 17]. Moving towards an agreed battery or sequence of tests for each relevant stage or step in the validation process would allow for much more informative comparison of validity and reliability findings.

This discussion incorporates free-living versus laboratory based comparisons. Feasibility and acceptability in the free living context may be more important than high statistical agreement in laboratory testing. Whilst debates have taken place on this issue in relation to the interpretation of findings [18, 19], this issue remains under-discussed in the majority of validity and reliability statements. We believe that a framework that emphasises the importance of all these judgements of feasibility and face, content, and context validity, and moves us beyond basic reporting of statistical values would be of great value to the field of PA and SB measurement.

To incorporate all the components of validity we need to consider, the term *context validity* has been proposed (see Table 2). This is an appealing idea, though the term has previously been coined in psychology in reference to interventions [20] so requires further thought.

**Table 2:** Methodological framework for establishing feasibility, validity and reliability

**Stage**	**Process**
Proof of concept–feasibility	1. Field testing and pilot testing of measure in controlled and free-living settings
Content and Face validity	2. Examination of relevant literature 3. Consultation with relevant experts 4. Theoretical examination of measure and domain/dimension 5. Examination of proposed data processing and decision algorithms including sensitivity analysis
Convergent validity	6. Assessment of the agreement between your measure and an existing (non-criterion) measure
Criterion validity	7. Assessment of the agreement between your measure and a criterion measure
Internal validity	8. Examination of bias such as reactivity and missing data
External validity	9. Examination of sample bias (age, sex, ethnic origin, socio-economic status)
Inter-rater reliability	10. Assessment of stability of tests administered by different researchers
Inter-instrument reliability	11. Assessment of stability of tests administered using multiple versions of the same instrument
Test-retest reliability	12. Assessment of stability of consecutive tests
Behavioural reliability	13. Assessment of stability accounting for behavioural changes
Context validity	14. Based on all assessments, will measure give useful information in the proposed context?
Purpose validity	15. Based on all assessments and considering study design, are the validity and reliability results suitable for the proposed use and likely to allow the research question to be answered?

#### Validity and reliability do not just depend on the PA or SB domain or dimension being assessed

The study design in which measurements will be taken is also fundamental [9, 21]. Different degrees of validity and reliability may be acceptable (or not) depending on whether the data are being used for population surveillance purposes, for the assessment of burden, risk or association, or to investigate intervention effects. Data from a measure may be valid and reliable in one type of study, and neither valid nor reliable enough in another (see Box 4). We suggest that validity and reliability for design is also reported in any considerations of validity and reliability.

We put these next ideas forward for debate: (1) if a measure is going to be used to stratify participants into 3 categories, perhaps this is how it should be validated–systematic bias is less important than the extent to which individuals are placed into the correct strata; (2) if the analysed outcome variable is dichotomous (e.g. active vs inactive), perhaps validation should be along the diagnostic lines of sensitivity and specificity (rather than the more usual agreement with a continuous measure of PAEE). This is perhaps where we should be discussing *relative validity* (i.e., the ability to correctly rank or compare people) rather than *absolute validity* (i.e., the ability to correctly quantify exposure).

We propose that a framework that emphasised intended study design when considering validity and reliability would improve the interpretation and use of PA and SB research. It may also help avoid situations where instruments designed for global assessment of PA or SB are used to detect individual level behaviour change, and the unfortunate rejection of interventions that may actually be working (or vice versa).

#### Reliability has two important constructs that are often misleadingly considered at the same time

The reliability of a measure of PA or SB will depend on two moving targets; (1) random error in the measurements (e.g., from changeable memory of activity within and between persons, or from intra- and inter-device variations); and (2) from real changes in the behaviour with time [21]. These real changes occur because very few people will conduct the exact same behaviour day after day. Therefore, a repeated measures design, which compares two time-points, is assessing the reliability of the behaviour and the measure at the same time. Studies with such designs often report the measures to have low reliability, rather than acknowledging that the behaviour itself might have low reliability or stability.

There are test-retest designs that can offer solutions when measuring unstable behaviour; behaviour can be known from a directed lab test of repeated 1000 steps, from directed sitting protocols, or from 24 h researcher observation. The change in behaviour can then be nullified or accounted for to isolate the reliability construct of interest.

This once again leads us to emphasise study purpose; if an epidemiological study wishes to assume PA status over a number of years, what sort of reliability tests are required to understand if the measurement period (and/or frequency) is appropriate? Alternatively, if an intervention wishes to understand how responsive their measure is, or how stable any observed changes are, what sort of reliability data will they need? An agreed framework for considering reliability that separates instrument stability from behavioural stability, and considers reliability timeframes, would clearly benefit PA and SB measurement.

While considering reliability we would also raise ways this can be assessed at a group level. If you measure each member of a group one time with your new measure and simultaneously with a criterion you can assess how random error varies between individuals (though not within) [22]. How this random error relates to our understanding of reliability and expectations of test-retest designs requires further examination.

#### We forget to discuss the validity of how data are processed and analysed

So far we have discussed validity and reliability in relation to measurements. However, we often have to make decisions about collected data to produce PA or SB outcome variables. For example, the huge variety of approaches for analysing accelerometer data has been noted with calls for consensus and standardised approaches [23]. Likewise, when dealing with self-reported data cycling may be counted as muscle strengthening, or a full 8 h per day of activity or sitting allocated when someone reports being active or sedentary at work [24]. Whether these decision algorithms provide valid outcome variables should be considered, potentially within face and content validity. Our proposed framework would emphasise examination of these considerations. It has been suggested that it is more appropriate to refer to validity and reliability of the obtained data, rather than the measurement instrument [21] and we would extend this to the PA or SB variables that are generated.

#### Terminology is used randomly, synonymously, possibly incorrectly and we all get confused

Already we have used terms that you may have taken issue with. In many places we could have used different terms such as precision, concordance, uncertainty, or accuracy. There are also many sub-types of validity and reliability, some of which we have not yet discussed. For example, construct, comparative, absolute, relative, predictive, discriminant, representation, and translation validity; and inter-rater, intra-rater, relative, or absolute reliability.

We suggest that this is because in PA and SB measurement we have largely borrowed our terms from older fields such as psychology, education, and nutrition. The field of PA and SB measurement has not yet agreed an accepted set of terms or appropriate utilization of these terms. Closely related fields such as physiology have attempted to codify these terms in a meaningful way [25] and we believe agreeing what these terms mean in the context of PA and SB measurement will help our science. In addition it would make teaching these concepts to our students much easier!

#### Evidence based medicine has benefited from existing frameworks and reporting guidelines

The evidence based medicine movement has introduced reporting guidelines to standardize and improve the way the evidence is presented and interpreted; from observational studies, to systematic reviews, to tools for assessing study bias these have been widely adopted [26] and are generally considered to have improved the scientific literature through greater comparability and transparency [27].

In the same way, we argue that a standardized approach to assessing, presenting and discussing validity and reliability would benefit our own field. There are existing approaches that we can build from; the MRC Diet and Physical Activity Assessment Toolkit provides a helpful glossary for certain types of validity and reliability [28]; the Social Research Methods Knowledge Base has a breakdown of terms with a slightly different use of terminology [29]; and Morrow has presented a useful schematic for how validity and reliability fit with terms such as objectivity and relevance in physiological assessment [25]. The MRC Toolkit has a Decision Matrix and Practical Consideration section which encourages readers to consider these issues as well as the “objectives of the research question” [28]. However, it does not explain how to consider these issues and offers dichotomous “green tick/no green tick” assessments of each consideration.

There are important existing papers to discuss. The COSMIN (COnsensus-based Standards for the selection of health status Measurement INstruments) checklist includes a taxonomy of terminology [30] and a checklist for considering different aspects of quality [9]. COSMIN was developed to assess subjective health-related patient-reported outcomes [9] and is designed for evaluating the quality of studies on measurement properties, rather than the concepts of validity and reliability in relation to measurement as our framework is intended [11]. While the COSMIN checklist is highly cited–we (non-systematically) found very few examples of use in PA for health and none in SB studies–this is perhaps due to its focus on clinical settings. Further, we disagree with aspects of their taxonomy e.g., measurement error under reliability [9, 30]; we consider measurement error to be comprised of random error which influences reliability and systematic error which influences validity. More focussed on PA and SB for health is the Hagströmer-Bowles Physical Activity-Sedentary-Behavior Questionnaire Checklist (HBQC) which is also designed to assess study quality in validation studies [31].

The Quality Assessment of Physical Activity Questionnaires (QAPAQ) checklist was developed from COSMIN [10]. It assesses what the authors term the qualitative attributes of questionnaires and is intended as a tool for selecting a PA questionnaire for a certain purpose as well as to design a measurement study [9]. Finally, Strath et al. have published a well cited and very helpful decision matrix for selecting PA assessment instruments which is very strong on feasibility considerations [32]. Our framework is not intended to supersede these, but proposes a preceding step in reconsidering the meaning and interpretation of validity and reliability in PA and SB measurement prior to applying these tools.

#### Finally, much of what we are about to propose is missed in validity and reliability statements

We have made the decision not to cite studies to support existence of the problems highlighted above, out of respect to colleagues, but also because we are guilty of this ourselves and need not go any further [22, 33–36]. It is also possible that with the multitude of validities and reliabilities available, the choice is overwhelming and researchers tend to use the general terms as a catch all; however, as we have argued this lack of clarity can lead to problems.

We hope that at least some of the arguments above have persuaded the reader that a framework for standardizing how we approach validity and reliability would be beneficial. Further, that single un-contextualized statements on validity and or reliability are at best unhelpful and at worst leading to misinterpretation of evidence on PA and SB.

### Part 2: the Edinburgh Validity and Reliability Framework

#### If a framework is needed, what should be in it?

We propose that not only do we need an agreed set of terminology and descriptions, but more importantly we need to understand how they should be used and related to make an overall meaningful assessment of validity and reliability. Here is our first attempt. We do not view this to be a complete, comprehensive, or static framework. Instead this version is presented to initially address the key issues outlined above, and subsequently stimulate debate leading to future iterations that incorporate the expert opinions of the field. As such, we have been selective in the concepts we have included. Connoisseurs of measurement theory may be disappointed to see we have omitted for example, nomological validity or discriminant validity in this first attempt. Selections were made on a pragmatic basis. We look forward to working on version 2.0 and beyond to include and amend all aspects that the research community deems necessary.

#### The Edinburgh Framework v1.0 for validity and reliability in PA and SB measurement

**Fig. 3:**
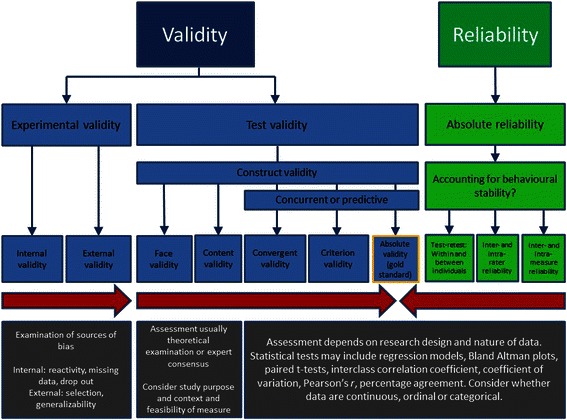
The Edinburgh Framework v1.0 for validity and reliability in PA and SB measurement

Figure 3 shows the schematic layout of the framework we propose. The terms used are briefly described in Table 1 to illustrate the different concepts and aspects and to allow debate and refinement. We fully expect that these need to be expanded and improved in respect to the context of PA and SB measurement. We acknowledge the MRC Diet and Physical Activity Measurement Tool Kit [28] and the Social Research Methods Knowledge Base [29] as important sources for this section amongst others. The red arrows in Fig. 3 are intended to illustrate the process and inter-relatedness of these concepts and show that assessments of validity and reliability should work across all aspects rather than individual aspects in isolation.

#### What needs further discussion?

We have omitted many important considerations from this initial list; for example *nomological*, *discriminant* and *divergent* validity and *relative* and *internal consistency* reliability [29]. Nor have we discussed how reliability influences validity; or how variable magnitudes of random error (unreliability) differentially affect the ability to detect valid mean values (or the number of repeat measurements required). There are also important questions about how we organise our proposed framework; for example is it fair to conflate construct and test validity; and perhaps internal and external validity are also constructs?

On the topic of external validity, further discussion is required about when to consider it and how much to weight these considerations. Many validity and reliability studies are conducted in University students and staff and we as guilty as anyone of this [33, 37]. While these can be useful proofs of concept, we may need better ways of understanding the generalizability of validity and reliability findings.

#### Using the Edinburgh Framework for establishing the validity and reliability in PA or SB measurement

Beyond simply describing different types of validity and reliability we want to change the way they are considered in PA and SB measurement. A methodological framework should provide a strategy or pathway for combining research methods and analytic techniques to answer a research question; in this case how valid and reliable are my PA or SB measures? The Edinburgh Framework emphasises that all aspects are important and provides a strategy for overall assessment. Only once all relevant aspects have been considered, the evidence reviewed, or measures empirically tested should statements about overall validity and reliability be made.

Table 2 depicts a possible way this process could work and be presented–we think that including this table (or a refined version) as a supplementary file in publications would improve the way we use, understand, and reflect on validity and reliability. It also suggests a process for validation studies to follow though the existing frameworks discussed already provide this [31]. One issue with existing frameworks and reporting guidelines is whether summary judgements are descriptive or quantified. We suggest a descriptive approach to avoid an overemphasis on quantifiable statistics that we have previously argued against (see point 4 above). We invite debate on how overall judgements could be presented.

#### Limitations

There are some obvious limitations with our proposal. We have not systematically examined the ideas presented. Whether you agree that some, none, or all these examples are self-evident and whether you agree they are of significant concern probably frames your stance on this debate. It is likely that a systematic study of the measurement literature would lead to an improved framework as it could identify and address the issues that could be demonstrated to be most common and important. Further, empirical demonstration of the utility of the framework is clearly missing from this debate.

We have only briefly described the methods for how each aspect of validity or reliability could be assessed. Ultimately, we envisage that future versions of the framework will include more options and guidance for selecting what aspects are important to assess. It could also be more instructive on the battery of tests (statistical or otherwise) available for each aspect, and importantly how to interpret the findings.

It is possible that such a framework places unnecessary extra burden on researchers for minimal gain. They already have many requirements and a lack of space when trying to publish findings. Perhaps the framework would be better suited as a format for validation and comparison of measures studies. Finally, this work is clearly directed at quantitative measurement. We make no claims about the use of these concepts in qualitative research.

#### Future work

Previous work by Mokkink et al., has demonstrated how systematic literature reviewing and Delphi methods with experts can generate consensus and produce useful tools for researchers [11]. We recommend that a similar process with experts in PA and SB for health measurement should be followed before any finalised framework could be proposed. Systematic literature review(s) will also help guide this work to the most relevant and prevalent challenges. In a recent review of wearable activity trackers Evenson et al., call for an evidence-based position statement on the properties necessary to consider a tracker valid and reliable [38]. We suggest that the work we propose could lead to such a position statement, but for all available methods (and the data they produce). The resulting framework and statements should focus on solutions to the challenges described in this article, so that researchers may have a clear pathway to better assessing validity and reliability. As suggested above, empirical assessment of the utility of any framework should also be conducted. It is fundamental to investigate if any finalised framework adds value, and is resource efficient, in our pursuit of better understanding PA and SB.

## Conclusion

In this debate we have argued that PA and SB measurement need a framework for considering the different aspects of measurement validity and reliability; we subsequently propose a draft version of such a framework. As stated we welcome critique, rebuttal, comment, and discussion on all ideas presented above. We hope the Edinburgh Framework provides a rationale and strategy for assessing any measures used in PA and SB measurement. We often find ourselves asking “what is the best measure (I can afford)” instead of asking “what can different measures tell us”? If we allowed ourselves to use a range of objective and subjective measures understanding their purpose and strengths, we may learn more about the complicated world of PA and SB.

## Box 1.

Accelerometers can be very good at detecting total movement over a given timeframe but may be less good at distinguishing types of PA. Therefore, to say they are a valid measure of PA without stating for which aspect(s) is a potentially misleading statement. Likewise, within single dimensions, measures may be differentially valid; a measure might be very good at detecting duration of walking or sitting, but unable to detect cycling or the difference between sitting watching TV and sitting working. That measure would therefore be of limited validity to give information about duration of all PA or SB.

## Box 2.

An active travel intervention might have a shift from passive to active transport modes as the most appropriate primary outcome; an intervention to break sedentary time might have fragmentation rate; an intervention to reduce falls may have bouts of increasing muscle strengthening activities. In all cases these are behaviours and should be assessed by a behavioural measure; while there may be some weak correlation to PAEE the success of the intervention and the choice of behavioural measure should not be guided by total energy expenditure.

## Box 3.

To test a questionnaire that is designed to detect frequency of a specific behaviour (e.g. bouts of walking) the selection of the comparator must be guided by the purpose of the questionnaire. While accelerometers are excellent movement sensors, they are currently not the best measures of walking bouts. Any comparison will include the measurement error in the accelerometer but almost always assign this as error in the questionnaire–this convergent validity would be potentially informative but not criterion. The validation should use methods that detect bouts of walking well such as GPS or direct observation.

## Box 4.

An epidemiological study that has many thousands of participants and intends to categorise PA or SB status (e.g. into low, medium or high; or meeting PA recommendations vs not meeting PA recommendations) requires a certain level of validity; it can handle a certain level of bias or random error due to the study design, likely sample size, and analysis approaches. In contrast if the study design is to detect change in SB of 50 participants at two time-points, it will be more concerned with reliability and random error than bias; it will need to have the desired responsiveness to detect change.

## Abbreviations

DLW: doubly labelled water
MET: metabolic equivalent of task
PA: physical activity
PAEE: physical activity energy expenditure
SB: sedentary behaviour

## References

[1] Rowe D (2011). Back to the future? Algorithms and equipment vs. simplicity and common sense in physical activity assessment. 23rd International Sport Science Congress, Daegu, Korea.

[2] Thompson D, Peacock O, Western M, Batterham AM (2015). Multidimensional physical activity: an opportunity, not a problem. Exerc Sport Sci Rev.

[3] Sedentary Behaviour Research Network (2012). Letter to the editor: standardized use of the terms “sedentary” and “sedentary behaviours”. Appl Physiol Nutr Metab.

[4] Chastin SFM, Schwarz U, Skelton DA (2013). Development of a consensus taxonomy of sedentary behaviors (SIT): report of Delphi round 1. PLoS One.

[5] Ainsworth BE, Haskell WL, Herrmann SD, Meckes N, Bassett DR, Tudor-Locke C (2011). 2011 Compendium of Physical Activities: a second update of codes and MET values. Med Sci Sports Exerc.

[6] Welk G (2002). Physical activity assessments for health-related research.

[7] Krizek KJ, Handy SL, Forsyth A (2009). Explaining changes in walking and bicycling behavior: challenges for transportation research. Environ Plann Plann Des.

[8] Atkin AJ, Gorely T, Clemes SA, Yates T, Edwardson C, Brage S (2012). Methods of measurement in epidemiology: sedentary behaviour. Int J Epidemiol.

[9] Terwee CB, Mokkink LB, van Poppel MN, Chinapaw MJ, van Mechelen W, de Vet HC (2010). Qualitative attributes and measurement properties of physical activity questionnaires: a checklist. Sports Med.

[10] van Poppel MN, Chinapaw MJ, Mokkink LB, van Mechelen W, Terwee CB (2010). Physical activity questionnaires for adults: a systematic review of measurement properties. Sports Med.

[11] Mokkink LB, Terwee CB, Knol DL, Stratford PW, Alonso J, Patrick DL (2010). The COSMIN checklist for evaluating the methodological quality of studies on measurement properties: a clarification of its content. BMC Med Res Methodol.

[12] Ainsworth BE, Caspersen CJ, Matthews CE, Mâsse LC, Baranowski T, Zhu W (2012). Recommendations to improve the accuracy of estimates of physical activity derived from self report. J Phys Act Health.

[13] Plasqui G, Bonomi AG, Westerterp KR (2013). Daily physical activity assessment with accelerometers: new insights and validation studies. Obes Rev.

[14] Prince SA, Adamo KB, Hamel ME, Hardt J, Gorber SC, Tremblay M (2008). A comparison of direct versus self-report measures for assessing physical activity in adults: a systematic review. Int J Behav Nutr Phys Act.

[15] Troiano RP, McClain JJ, Brychta RJ, Chen KY (2014). Evolution of accelerometer methods for physical activity research. Br J Sports Med.

[16] Hopkins WG. Bias in Bland-Altman but not regression validity analyses. Sportscience. 2004;8(4). http://www.circ.ahajournals.sportsci.org/index.html.

[17] Bland JM, Altman D (1986). Statistical methods for assessing agreement between two methods of clinical measurement. Lancet.

[18] Ekelund U, Brage S, Wareham NJ (2004). Physical activity in young children. Lancet.

[19] Reilly JJ, Jackson DM, Paton JY (2004). Physical activity in young children. Lancet.

[20] Skinner CH. Contextual validity: knowing what works is necessary, but not sufficient. Display Ad Rates. 2013;15.

[21] Masse LC, de Niet JE (2012). Sources of validity evidence needed with self-report measures of physical activity. J Phys Act Health.

[22] Kelly P, Doherty A, Mizdrak A, Marshall S, Kerr J, Legge A (2014). High group level validity but high random error of a self-report travel diary, as assessed by wearable cameras. J Transp Health.

[23] Wijndaele K, Westgate K, Stephens SK, Blair SN, Bull FC, Chastin SF (2015). Utilization and harmonization of adult accelerometry data: review and expert consensus. Med Sci Sports Exerc.

[24] Corbett J, Day J, Doig M, Dowling S, Martin S, Stannard A (2014). The Scottish health survey 2013. Volume 2: technical report.

[25] Morrow JR (2011). Measurement and evaluation in human performance.

[26] Simera I, Moher D, Hirst A, Hoey J, Schulz KF, Altman DG (2010). Transparent and accurate reporting increases reliability, utility, and impact of your research: reporting guidelines and the EQUATOR Network. BMC Med.

[27] Chan EH, Zumbo BD, Chan EKH (2014). Standards and guidelines for validation practices: development and evaluation of measurement instruments. Validity and validation in social, behavioral, and health sciences.

[28] Medical Research Council. Diet and physical activity measurement toolkit (accessed 1/10/15). http://dapa-toolkit.mrc.ac.uk/. Accessed 1/10/15

[29] Social Research Methods. Research Methods Knowledge Base (accessed 1/10/15). http://www.socialresearchmethods.net/kb/index.php. Accessed 1/10/15.

[30] Mokkink LB, Terwee CB, Patrick DL, Alonso J, Stratford PW, Knol DL (2010). The COSMIN study reached international consensus on taxonomy, terminology, and definitions of measurement properties for health-related patient-reported outcomes. J Clin Epidemiol.

[31] Hagstromer M, Ainsworth BE, Kwak L, Bowles HR (2012). A checklist for evaluating the methodological quality of validation studies on self-report instruments for physical activity and sedentary behavior. J Phys Act Health.

[32] Strath SJ, Kaminsky LA, Ainsworth BE, Ekelund U, Freedson PS, Gary RA (2013). Guide to the assessment of physical activity: clinical and research applications: a scientific statement from the American Heart Association. Circulation.

[33] Kelly P, Doherty A, Berry E, Hodges S, Batterham AM, Foster C. Can we use digital life-log images to investigate active and sedentary travel behaviour? Results from a pilot study. Int J Behav Nutr Phys Act. 2011;8. doi:10.1186/1479-5868-8-44 10.1186/1479-5868-8-44 PMC3118309 21599935

[34] Kelly P, Doherty AR, Hamilton A, Matthews A, Batterham AM, Nelson M (2012). Evaluating the feasibility of measuring travel to school using a wearable camera. Am J Prev Med.

[35] Kelly P, Krenn P, Titze S, Stopher P, Foster C (2013). Quantifying the difference between self-reported and global positioning systems-measured journey durations: a systematic review. Transp Rev.

[36] Baker G, Gray SR, Wright A, Fitzsimons C, Nimmo M, Lowry R (2008). The effect of a pedometer-based community walking intervention “Walking for Wellbeing in the West” on physical activity levels and health outcomes: a 12-week randomized controlled trial. Int J Behav Nutr Phys Act.

[37] Kelly P, Thomas E, Doherty A, Harms T, Burke Ó, Gershuny J (2015). Developing a method to test the validity of 24 hour time use diaries using wearable cameras: a feasibility pilot. PLoS One.

[38] Evenson KR, Goto MM, Furberg RD (2015). Systematic review of the validity and reliability of consumer-wearable activity trackers. Int J Behav Nutr Phys Act.

